# Von Hippel-Lindau Syndrome: Diagnosis and Management of Hemangioblastoma and Pheochromocytoma

**DOI:** 10.1155/2013/624096

**Published:** 2013-05-23

**Authors:** P. Vaganovs, K. Bokums, E. Miklaševics, J. Plonis, L. Zarina, I. Geldners, J. Gardovskis, E. Vjaters

**Affiliations:** Clinic of Urology, Pauls Stradins Clinical University Hospital, Pilsonu Street 13, Riga Latvia, LV-1002, Latvia

## Abstract

*Introduction*. Von Hippel-Lindau (VHL) syndrome is a pathological condition that causes various clinical symptoms and is difficult to diagnose. The most common pathological lesions are hemangioblastomas of the central nervous system, retinal angiomas, renal clear cell carcinomas, and pheochromocytomas. *Case Report*. A 23-year-old female had a syncope episode in 2008. Magnetic resonance imaging (MRI) revealed a right temporal hemangioblastoma, which was treated surgically. Genetic screening identified a VHL gene mutation, and computed tomography (CT) revealed a left adrenal mass. Since it was unclear whether the mass was a pheochromocytoma, or another benign or malignant tumors, laparoscopic adrenalectomy was performed. A month after surgery, the patient complained of general fatigue, poor concentration, loss of appetite, and insomnia. After careful clinical investigation, the patient was referred to a psychiatrist due to suspected depression, which was confirmed. *Conclusions*. VHL genetic screening should be performed in cases of hemangioblastoma. In VHL syndrome cases, pheochromocytoma cannot always be diagnosed by biochemical catecholamine analyses; therefore, CT or MRI scanning of the abdomen must be performed. Due to the long treatment period, some patients may develop episodes of depression, which can simulate VHL syndrome.

## 1. Introduction

Von Hippel-Lindau (VHL) syndrome is a rare hereditary cancer characterized by benign or malignant tumors. The most common VHL syndrome-associated tumors are hemangioblastomas of the central nervous system, pheochromocytomas, retinal angiomas, neuroendocrine tumors, clear cell renal carcinomas, and middle ear tumors that affect the endolymphatic sac [1]. The current clinical classification of VHL comprises three types, one of which has three subtypes. Type 1 is characterized by a high risk of renal cell carcinoma and a low risk of pheochromocytoma. Types 2a and 2b are characterized by a high risk of both renal cell carcinoma and pheochromocytoma. Type 2c is characterized by a high risk of pheochromocytoma, whereas type 3 is characterized by a risk of Chuvash polycythemia [2]. VHL develops in childhood or adolescence, is a rare condition presented in age over 60, and is autosomal dominant [3]. Since the syndrome expresses itself at such young age, VHL is one of the most investigated and well-reported hereditary conditions. Several authors have sought to classify the clinical manifestations of VHL syndrome according to the presence of VHL mutations. The VHL gene is located on chromosome 3p25, consists of three exons [4], and produces at least two isotypes of pVHL (a tumor suppressor protein). pVHL mainly acts by directly regulating the levels of hypoxia-inducible transcription factor types 1 and 2 (HIF1 and HIF2), which play important roles in the cellular response to oxygen deficiency. When pVHL is not produced due to VHL gene mutations (mostly deletions), HIF is not blocked; this promotes angioneogenesis and uncontrolled cell proliferation. Thus, pVHL mutations seriously increase the risk of tumor growth in target organs. 

## 2. Case Presentation

**Figure 1:**
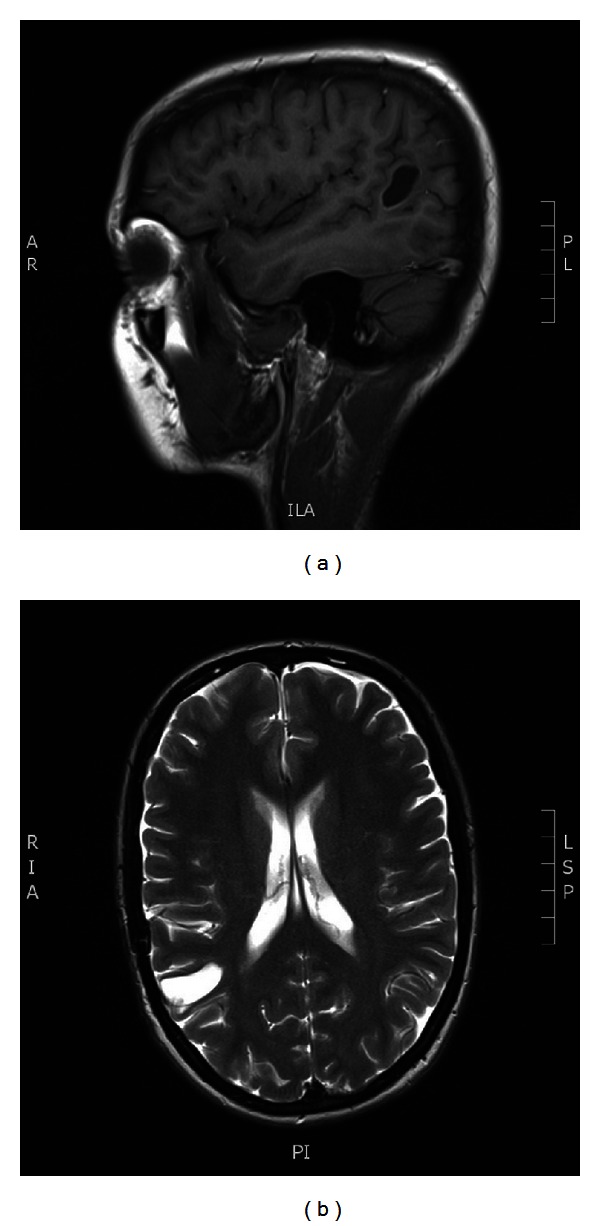
CT scan showing a hemangioblastoma in the right temporal lobe in the noncontrast and contrast series.

A 19-year-old woman was first admitted to the university hospital emergency department in September, 2008 after an episode of syncope. The patient consulted a neurologist, and blood analysis, biochemical analyses, and an MRI investigation of the brain were performed. These tests revealed a mass in the right temporal lobe (Figures 1(a) and 1(b)). The surgical removal of the hemangioblastoma went well, and there were no complications during the postoperative period. The clinical findings were confirmed histologically (hemangioblastoma), and the patient was diagnosed with suspected VHL syndrome. She was discharged in June, 2008.

**Figure 2:**
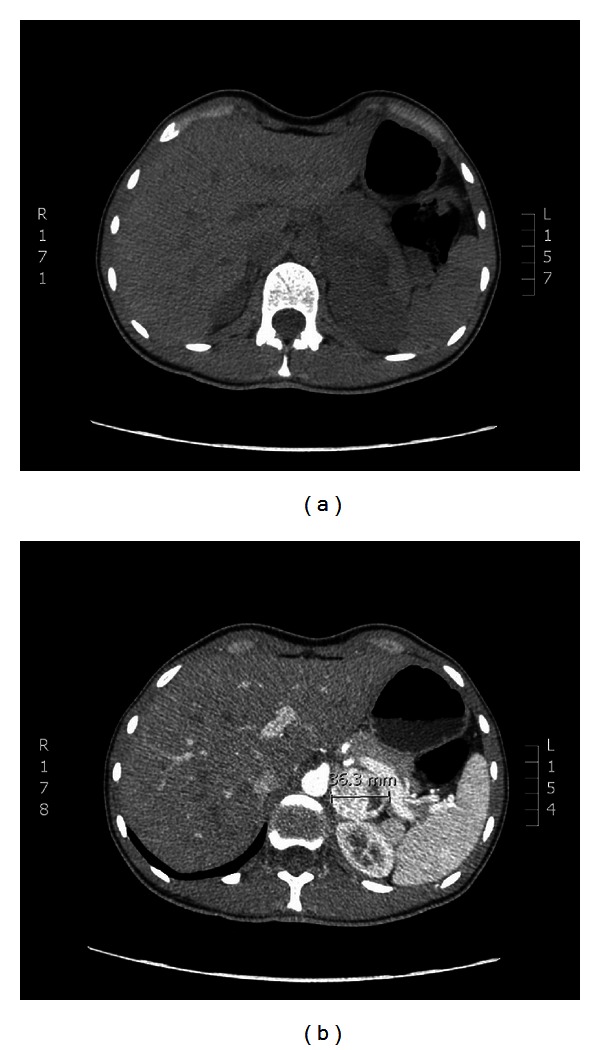
CT scan of the abdomen showing a mass in the left adrenal gland in the noncontrast and contrast series.

The patient was admitted to the university hospital emergency department for the second time in April 2012. This time, she presented with weakness, palpitations, and nausea, and an abdominal CT was performed both with and without contrast (Figures 2(a) and 2(b)). While the right adrenal gland did not show any pathological changes in the noncontrast images, a round, well-demarcated mass was observed in the left adrenal gland, which was about 36.3 mm in diameter. However, administration of the contrast reagent showed the mass to be hypervascular. The vascularization was homogeneous. In addition, the density of the mass increased up to 170 Hounsfield Units (HU). The contrast was leached in 15 minutes and the density decreased to 70 HU. There were no other pathological findings in the abdomen. The conclusion was that there was a mass in the left adrenal gland that was probably benign in nature (the leaching of the contrast exceeded 50%). However, these findings, along with the clinical signs of VHL disease, meant that it was still possible that the mass was a pheochromocytoma, which could have been dormant at that time but could, upon being triggered, release massive amounts of catecholamine. 

To exclude the possibility of lesions in other locations, MRI of the brain and a chest CT were performed. No compelling indications of pathology were found.

The patient was admitted to the endocrinology department, where catecholamine tests were performed. There were no deviations in catecholamine levels in daily urine or blood samples. Two days later, the patient was referred to a consilium, which decided that the patient's medical and family histories, along with the CT findings, warranted surgical intervention. The main indications for surgery were as follows: it was not possible to exclude the possibility that the current mass in the adrenal gland was a pheochromocytoma, and the mass was large (36.3 mm in diameter). Moreover, despite the radiologist's conclusion that the tumor was probably benign, there was still a possibility that it would progress and become malignant in the future.

**Figure 3:**
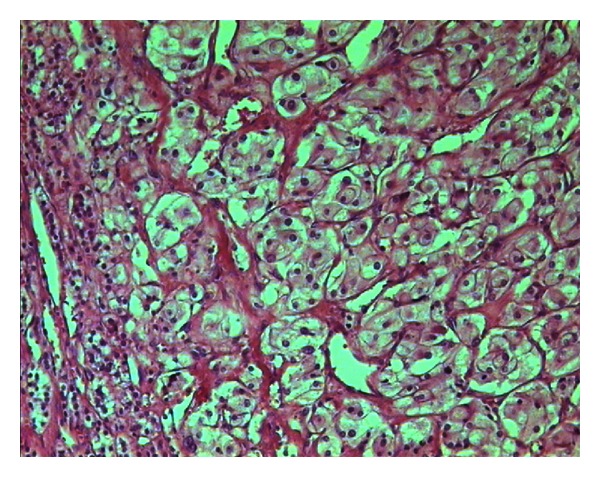
The lobulated hypercellular tumor is composed of large, polygonal cells with a low nuclear/cytoplasmic ratio and a grainy basophilic cytoplasm. The tumor is encircled by cells that form the cortical region of the adrenal gland. The mass is well demarcated, with no capsule or pseudocapsule.

**Figure 4:**
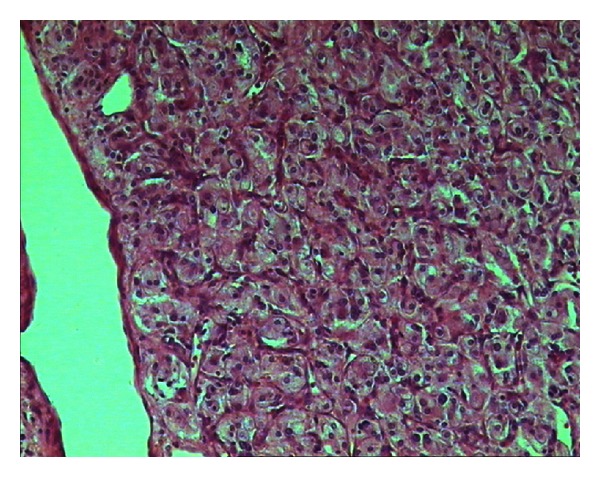
There is no tumor cell mitosis. There is no necrosis, no significant pleomorphism of the cells or their nuclei (including spindle-shaped cells, hyperchromasia of the nuclei, and large nucleoli), no tumor infiltration of the adrenal gland cortical tissues, no vascular or perineural invasion, and no invasion into the adrenal gland capsule (including the periadrenal tissues).

**Figure 5:**
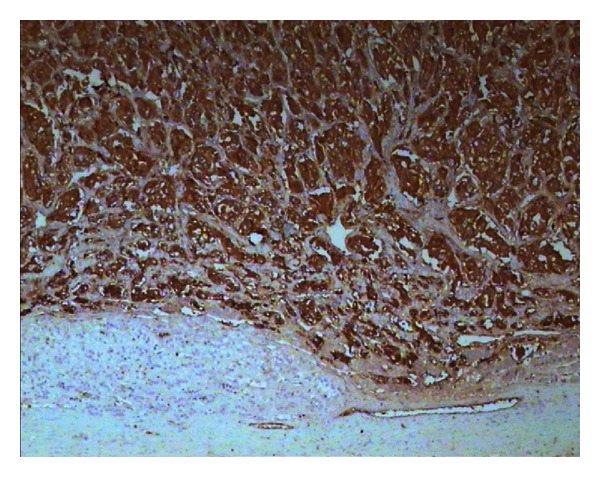
Diffuse cytoplasmic staining of tumor cells with an antibody specific for chromogranin A. Immunoperoxidase stain; mag ×40.

**Figure 6:**
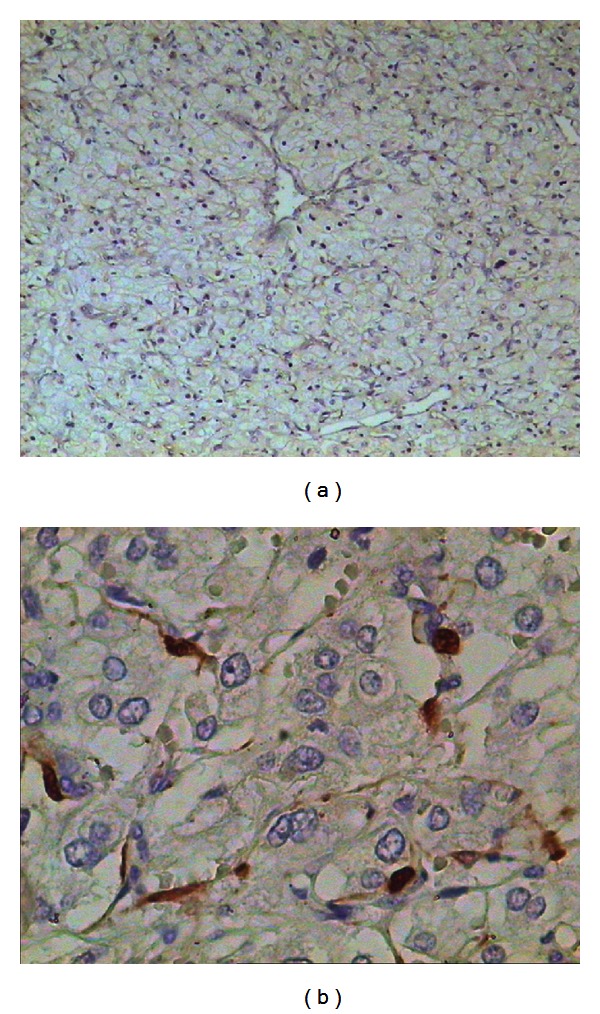
The proliferation index of Ki-67 (clone MIB-1) detection in the tumor is very low (<1%).

**Figure 7:**
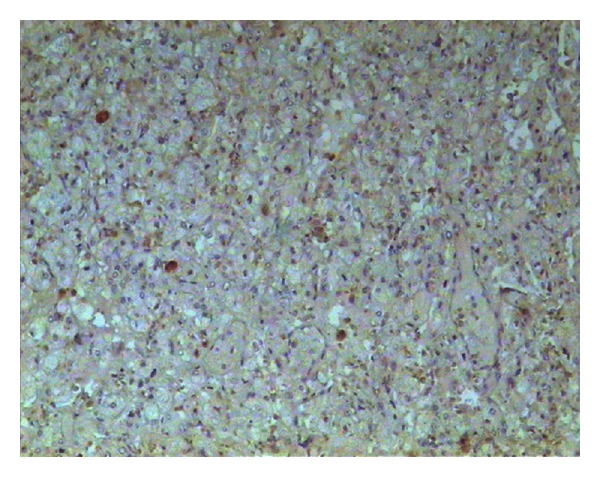
The tumor cells are negative for calretinin.

A left side laparoscopic adrenalectomy was performed 1 month after the initial presentation, followed by pathohistological investigations to confirm the diagnosis. Examination of standard histological hematoxylin and eosin-stained samples showed that the tumors in the adrenocortex were morphologically similar to pheochromocytoma. For this reason, and because of the absence of clinical signs typical of pheochromocytoma, the tumor was subjected to further immunohistochemical analyses (Figures 3, 4, 5, 6, and 7). The final pathohistological diagnosis was pheochromocytoma of the left adrenal gland.

The patient, her maternal grandmother, her maternal grandfather, and her uncle (the brother of the patient's mother) all underwent VHL gene sequencing. The patient was diagnosed with a genetic mutation in the VHL gene; however, the grandparents and the uncle did not harbor mutations. Genetic consultation confirmed a diagnosis of VHL type 2a. To date, the patient does not agree to the screening of her 12 years old son's VHL gene. 

**Figure 8:**
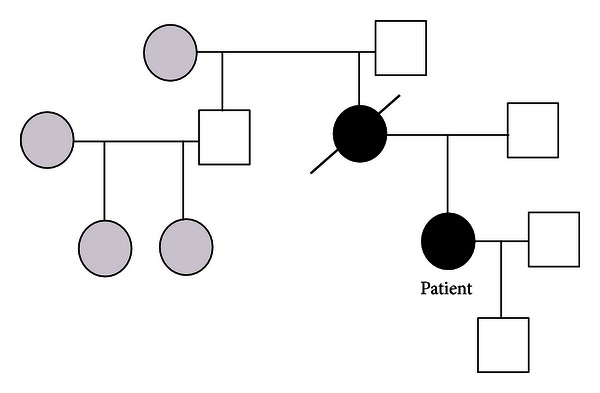
Genealogical tree.

During the enquiry about the family history (Figure 8), it was revealed that the patient's mother had died from an intracerebral hemorrhage at the age of 41. The patient's mother past medical history revealed that she suffered recurrent hemangioblastomas in multiple areas, namely, the right optical nerve, the posterior fossa, and the cervical section of the spinal cord. Moreover, she had pheochromocytomas, including ectopic pheochromocytomas. The patient's mother was cured after resection of the hemangioblastoma and a bilateral adrenalectomy. Given that the grandparents and the uncle did not harbor VHL mutations, it is possible that the mutation occurred in the grandfather during spermatogenesis. This is supported by the fact that the grandfather served on a Soviet nuclear submarine during his adolescence. 

Approximately 1 month after surgery, the patient complained about general fatigue, poor concentration, loss of appetite, and insomnia. The patient was examined by a physician, but laboratory testing revealed no abnormalities. During the next 2 weeks, the patient's symptoms worsened, and she was referred to a psychiatrist. The psychiatrist suspected depression and prescribed a selective serotonin reuptake inhibitor (SSRI). One month later, the patient's symptoms had improved; however, she continued to complain of morning fatigue and a loss of interest in daily activities, which are classic symptoms of depression. 

Four months after the patient first visited the psychiatrist, she remains under psychiatric supervision for symptoms of depression. At her last appointment in the urological outpatient department, she complained that her daily activities gave her no pleasure and that her appetite had increased over the last week. Her surgical scar looks good, and the patient has no physical problems. 

## 3. Discussion

Since VHL syndrome is not a common disorder (it occurs in approximately 3 : 100 000 births per year) and is associated with a wide spectrum of symptoms, early diagnosis can be difficult [3]. The wide spectrum of symptoms means that VHL syndrome is divided into two different phenotypes, the second of which is further divided into three subtypes. Type 2a is associated with a lower incidence of renal cell cancer, whereas VHL type 2b is associated with a high incidence of pheochromocytoma and hemangioblastoma. 

The VHL mutation screening for the patient's son remains a matter for discussion, which is complicated by legal issues. However, early screening and identification of a VHL mutation would allow her son to be managed appropriately, which would include prophylactic CT scans of the head and abdomen. At present, VHL patients can only be treated according to their symptoms. 

The most appropriate management of hemangioblastoma is unclear. Patients with inoperable hemangioblastoma, or whose hemangioblastomas might be associated with a high operative complication rate, can be managed with conventional fractioned radiation therapy or stereotactic radiosurgery, which shows good results. One study suggests that small hemangioblastomas can be managed conservatively with radiological followup and that only symptomatic hemangioblastomas should be managed operatively [5]. The operative results are good, with no reported recurrences after surgery. However, there is a high risk of developing other hemangioblastomas in other areas of the central nervous system. Over the last decade, several reports have suggested that preoperative embolization of hemangioblastomas is feasible; this procedure decreases blood loss during surgery and reduces the incidence of postoperative complications [6]. In the present case, MRI was used to investigate the tumor, which led to surgical intervention. Before surgery, the neurosurgeons and the neuroradiologist discussed the possibility of preoperative embolization; however, due to the size and location of the hemangioblastoma, conservative surgery was performed without preoperative embolization.

There is a strong association between pheochromocytoma and VHL syndrome, and pheochromocytoma is an important feature in the clinical classification of VHL syndrome [7]. It usually develops in young adults, and its location can be either adrenal or extra-adrenal. Since pheochromocytomas can have low activity, the classical symptoms (such as tachycardia, diaphoresis, postural hypotension, tachypnea, cold and clammy skin, severe headache, angina, palpitations, nausea, vomiting, or epigastric pain) may be missing. Indeed, a study of 33 patients with VHL syndrome and 37 with pheochromocytomas showed that approximately 35% of patients did not have any clinical signs [7]. 

The absence of symptoms can make it difficult to diagnose pheochromocytoma. Indeed, in the present case, the classical symptoms of pheochromocytoma were absent. However, due to the risk of pheochromocytoma, CT scanning was performed, and an adrenal mass was detected. The mass could have been a pheochromocytoma or a malignant lesion. A diagnosis of pheochromocytoma is based on both biochemical and radiological confirmations. Plasma epinephrine or norepinephrine may be detected in the blood; however, vanillylmandelic acid must be detected in the urine [8]. On the basis of the biochemical results, radiological investigations (including abdominal CT or MRI scans) are performed to diagnose pheochromocytoma. In the present case, catecholamines were not detected in the blood or urine. Nevertheless, a prophylactic CT scan was performed due to the high possibility of asymptomatic pheochromocytoma. 

Several studies suggest that surgical resection is the best way to treat pheochromocytoma in patients with VHL syndrome; this approach shows excellent results, and no recurrences have been reported [9, 10]. Several studies also report that preoperative preparation should include the administration of alpha-adrenergic blockers, calcium channel blockers, or a combination of alpha- and beta-adrenergic blockers [11, 12]. Although the appropriate preoperative management remains under discussion, in our experience, administering an alpha-adrenergic blocker for 2 to 3 weeks preoperatively normalizes blood pressure and decreases the risk of operative complications. However, the current patient did not show any manifestations of pheochromocytoma. For this reason, she did not undergo preoperative treatment with antihypertensive drugs. In terms of surgery, laparoscopic or conventional approaches can be used. Over the last decade, laparoscopic adrenalectomy has become the gold standard. Our hospital generally performs laparoscopic adrenalectomy if there are no contraindications to laparoscopic surgery. In our opinion, this patient was a good candidate for laparoscopic adrenalectomy as the risk of complications during surgery was low.

## 4. Conclusions

VHL syndrome is often difficult to diagnose because of the wide range of symptoms. Patients diagnosed with hemangioblastoma should be tested for VHL gene mutations. If mutations are present, the patient's relatives should be offered a genetic consultation. In most countries, legislation demands that the parents or legal guardians must make decisions regarding the treatment of minors. Ethical problems regarding the screening of minors must be weighed against the clinical benefits of early diagnosis. CT or MRI scanning of the abdomen should be performed when catecholamine tests are normal. Due to the need for long-term treatment, patients with VHL are at risk of developing psychological and psychiatric conditions, which themselves can simulate somatic disorders.
